# Easily Regenerated Readily Deployable Absorbent for Heavy Metal Removal from Contaminated Water

**DOI:** 10.1038/s41598-017-06734-7

**Published:** 2017-07-27

**Authors:** Perry N. Alagappan, Jessica Heimann, Lauren Morrow, Enrico Andreoli, Andrew R. Barron

**Affiliations:** 1 Department of Chemistry, Rice University, Houston, Texas 77005 USA; 2 Energy Safety Research Institute, Swansea University, Bay Campus, Swansea, SA1 8EN Wales UK; 3 Department of Materials Science and Nanoengineering, Rice University, Houston, Texas 77005 USA

## Abstract

Although clean and abundant water is the keystone of thriving communities, increasing demand and volatile climate patterns are depleting rivers and aquifers. Moreover, the quality of such water sources is threatened by noxious contaminants, of which heavy metals represents an area of growing concern. Recently, graphene oxide (GO) has been suggested as an adsorbent; however, a support is desirable to ensure a high surface area and an immobile phase. Herein, we described the preparation and characterization of a supported-epoxidized carbon nanotube (SENT) via the growth of multi walled carbon nanotubes (MWNTs) onto a quartz substrate. Subsequent epoxidation provides sufficient functionality to enable adsorbent of heavy metals (Cd^2+^, Co^2+^, Cu^2+^, Hg^2+^, Ni^2+^, and Pb^2+^) from aqueous solution with initial concentrations (60–6000 ppm) chosen to simulate high industrial wastewater contamination. The SENT adsorption efficiency is >99.4% for all metals and the saturation concentration is significantly greater than observed for either GO or acid treated MWNTs. The SENT adsorbent may be readily regenerated under mild conditions using a globally available household chemical, vinegar. 1 g of SENT has the potential to treat 83,000 L of contaminated water down to WHO limits which would be sufficient for 11,000 people.

## Introduction

The lack of simple, effective, remediation technologies is a significant hindrance to the quality of living in developing regions of the world. One class of inorganic pollutants that is of particular concern is heavy metals because they have the ability of dissolving in wastewaters and when discharged into surface waters, they can be concentrated and travel up the food chain or seep into groundwater, hence contaminating drinking water^1, 2^. There are basically five sources of heavy metals contributing to water pollution: geological weathering (providing the background level); processing of ores and metals; use of metals and metal compounds; leaching of heavy metals from domestic wastes and solid waste dumps; and heavy metals in human and animal excretions^3^. Each of the existing technologies for removal (chemical precipitation, ion exchange, phytoremediation, and microbial remediation) has its merits and demerits^4–6^; however, there is a need for a simple process that can be deployed anywhere for treatment of drinking water. An ideal treatment system should be low cost with regard to its manufacture, raw materials and use. It should be easily deployed for any skill level and most importantly it must be able to be reused. The latter is an important consideration for implementation in developing regions.

Graphene oxide (GO) has been shown to be an efficient adsorbent of a range of metals^7, 8^. Unfortunately, its application relies on coagulation and subsequent filtration. In order to overcome this, magnetic nanoparticles have been attached to allow for magnetic separation of the contaminated GO from the water^8, 9^, but this leads to another level of complexity. The disposal of the contaminated GO is also potentially problematic, and in its use for drinking water the potential contamination by nanocarbon would be unacceptable^10^. As such, a supported form of a nanomaterial is desirable to ensure a high surface area and potentially be used as an immobile phase^11^. The uptake of various metal ions by GO was shown by Wang and co-workers^8, 12, 13^ to be due to the presence of oxygen functionality (including, hydroxyl, epoxide, carboxyl and carbonyl). Given that such functionality is predominantly concentrated on the edges of the graphene sheets, there is a limit to the O:C ratio and hence uptake efficiency^14^. We have shown single walled carbon nanotubes (SWCNTs) may be oxidized by simple chemical routes to give high concentrations of epoxide moieties^15^, suggesting that compared to GO, epoxidized-CNTs would be suitable adsorbents for heavy metals. Finally, we have recently reported the high yield synthesis of multi walled carbon nanotubes (MWCNTs) from a wide range of hydrocarbon sources using simple apparatus and low cost catalysts that can be adapted to manufacture in low tech environments^16^, which given the high cost of SWCNTs led us to investigate the creation (and epoxidation) of a supported MWCNT conjugate.

In this paper, we develop the growth of MWCNTs on to quartz wool support and their subsequent purification and epoxidation to form an efficient supported-epoxidized carbon nanotube (SENT) adsorbent of heavy metals from aqueous solution that may be readily regenerated under mild conditions using globally available household chemicals.

## Results

**Figure 1:**
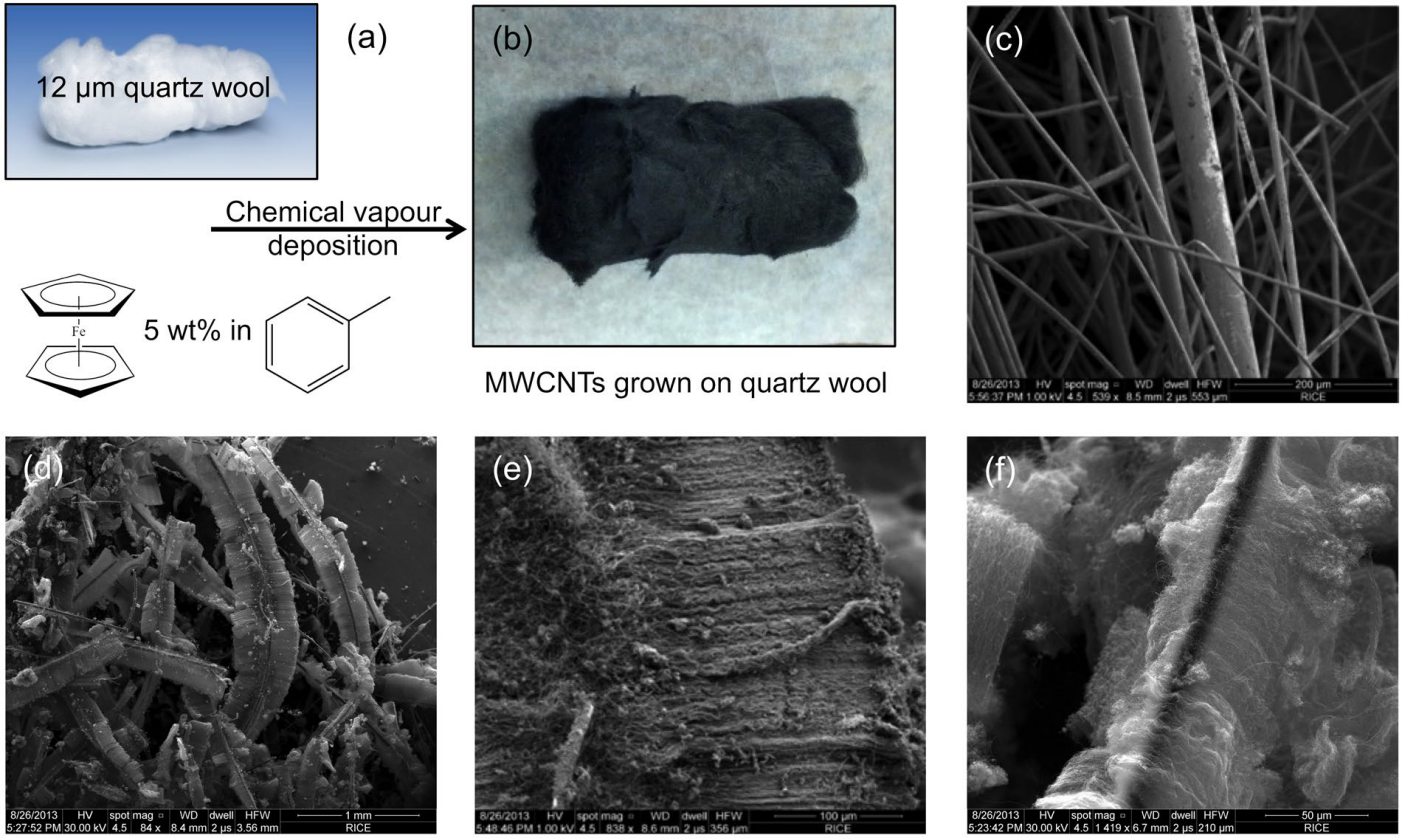
Images of the quartz wool supported-CNTs. (**a**) Schematic of the reaction for the growth of CNTs on to the quartz wool support showing (**b**) an optical image of the as grown quartz wool supported CNTs. SEM micrographs of (**c**) the quartz wool substrate, (**d** and **e**) the as grown quartz wool supported CNTs and (**f**) the supported epoxidized CNTs (SENTs).

Carbon nanotubes (CNTs) were grown (@ 900 °C) onto a quartz wool substrate using toluene as the carbon source and ferrocene as the catalyst precursor (Fig. 1a)^16^. The as-growth yield was generally in the range 80–90% with respect to the carbon precursor. The wool was “visually dark” throughout its length suggesting a uniform deposition density (Fig. 1b). SEM images of the as grown CNTs confirms the uniform growth over each quartz fibre (Fig. 1d); however, the length of the CNTs varies between 100–250 μm length (Fig. 1d and f) depending on the position within the reactor. The arrangement of the CNTs on the individual quartz fibres is reminiscent of two opposing brushes oriented either side of the quartz fibre (Fig. 1d): a tri-lobe structure of this type has been reported^17^. Closer inspection indicates that the orientation is a consequence of the inter-tube forces since the CNTs grow around the entire circumference of the quartz fiber, rather on two sides (Fig. 1f).

**Figure 2:**
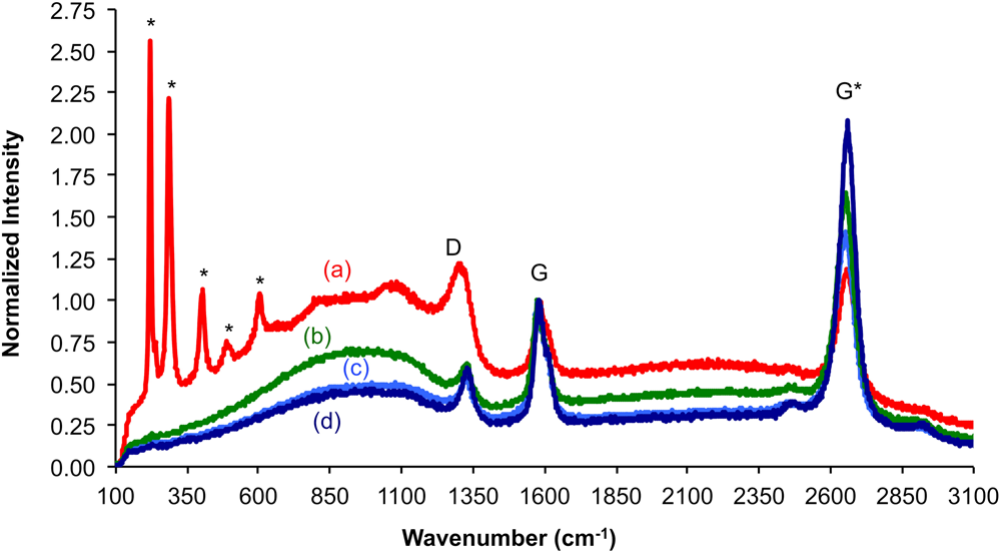
Raman spectra of the quartz wool supported-CNTs. Normalized Raman intensity (633 nm) of (**a**) as grown quartz wool supported CNTs showing peaks (*) due to iron oxide catalyst residue, which are removed after wet air oxidation/acid wash (**b**). The G:D ratio decreases upon epoxidation (**c**) due to functionalization of the CNT side walls, while adsorption of Cd^2+^ does not significantly effect the spectra (**d**), suggesting that adsorption occurs predominantly via the epoxide oxygen, rather than the CNT sidewall.

Thermogravimetric analysis (TGA) of CNTs grown under identical conditions in the absence of the quartz wool shows a 9.83 wt.% residue above 800 °C consistent with the presence of iron catalyst impurities. Purification by wet air oxidation (WAO) and acid wash^18^ resulted in a reduction of the residue to 4.68 wt.%. WAO treatment on the quartz wool supported CNTs demonstrated no perceptible change in the appearance; however, comparison of the G peak and D peak intensities in the Raman spectroscopy shows a clear effect (Fig. 2). The G mode are associated with tangential displacement C-C bond stretching motions (1500–1600 cm^−1^ range), while the D, or disorder mode (1290–1330 cm^−1^ depending on the Raman excitation laser wavelength), originates from crystallinity disorders and lattice imperfections and represents the presence^19^ and distribution^20^ of sp^3^ carbon centres. The G’ corresponds to disorder induced carbon features arising from finite particle size distribution or lattice distortion. The non-nanotube peaks in the Raman spectra of the as grown quartz wool supported CNTs (peaks marked with * in Fig. 2) match with hematite (Fe_2_O_3_)^21^ and lepidocrite (γ-FeO(OH)^22^. The peaks associated with iron oxide (catalyst residue) are absent after WAO. In addition the G:D ratio increases from 0.86 to 1.92 after WAO. This is consistent with the SEM images after purification that shows the removal of debris (Fig. 1f).

Epoxidation of the supported CNTs is accomplished using 3-chloroperoxybenzoic acid (*m*-CPBA). Raman analysis after epoxidation (Fig. 2) showed a slight decrease in the G:D ratio to 1.59 from 1.92. The decrease in the ratio after the epoxidation is expected because the addition of the epoxide groups to MWNTs slightly disrupts the graphitic structure^15^. TGA of analogous CNTs grown in the absence of the quartz wool shows a 2.74 wt.% residue above 800 °C consistent with functionalization of the CNTs.

Un-supported epoxidized CNTs were initially tested for metal sorption by addition to solutions of an excess of the appropriate metal (Zn^2+^, Co^2+^, Ni^2+^, and Cd^2+^) salts. TGA values for the metal saturated epoxidized CNTs showed between 3.1 and 9.5 wt.% increase in the residue consistent with metal adsorption. X-ray photoelectron spectra (XPS) confirm the presence of the appropriate metal. There was only a small change in the G:D ratio after metal adsorption (1.69 versus 1.59), suggesting that the epoxide group alone is responsible for adsorbing the metal.

Quantification of metal (Cd^2+^, Co^2+^, Cu^2+^, Hg^2+^, Ni^2+^, and Pb^2+^) uptake was determined by passing a standard aqueous solution of each metal through a known mass of SENTs in a burette. The initial concentrations (C_i_) for each metal (60–6000 ppm) was chosen to simulate cases of high industrial wastewater contamination (Table 1)^23^. The procedure was then repeated for 3 trials per metal. The residual concentration of metal (C_f_) was determined, by UV-visible spectroscopy, for each aliquot after passing through the SENT from which the adsorption efficiency (Eq. 1) for each metal compound was calculated (Table 1).

1 $${\rm{a}}{\rm{d}}{\rm{s}}{\rm{o}}{\rm{r}}{\rm{p}}{\rm{t}}{\rm{i}}{\rm{o}}{\rm{n}}\,{\rm{e}}{\rm{f}}{\rm{f}}{\rm{i}}{\rm{c}}{\rm{i}}{\rm{e}}{\rm{n}}{\rm{c}}{\rm{y}}\,({\rm{ \% }})=[1{\textstyle \text{-}}{(C}_{{\rm{f}}}{/C}_{{\rm{i}}})]\times 100$$

**Table 1:** Metal concentration and adsorption efficiency.

Metal compound	Initial concentration, mol/L (ppm)	Adsorption efficiency (%)	Saturation concentration, (mg M/g SENT)
cadmium (II) acetate	5.20 × 10^−2^ (5855)	99.38	581
cobalt (II) chloride	1.00 × 10^−3^ (60)	99.72	41.1
copper (II) sulphate	1.57 × 10^−3^ (100)	99.65	70.3
mercury (II) chloride	1.00 × 10^−2^ (2000)	99.61	199
nickel (II) chloride	1.00 × 10^−2^ (590)	99.70	463
lead (II) acetate	4.83 × 10^−4^ (100)	99.97	69.8

**Figure 3:**
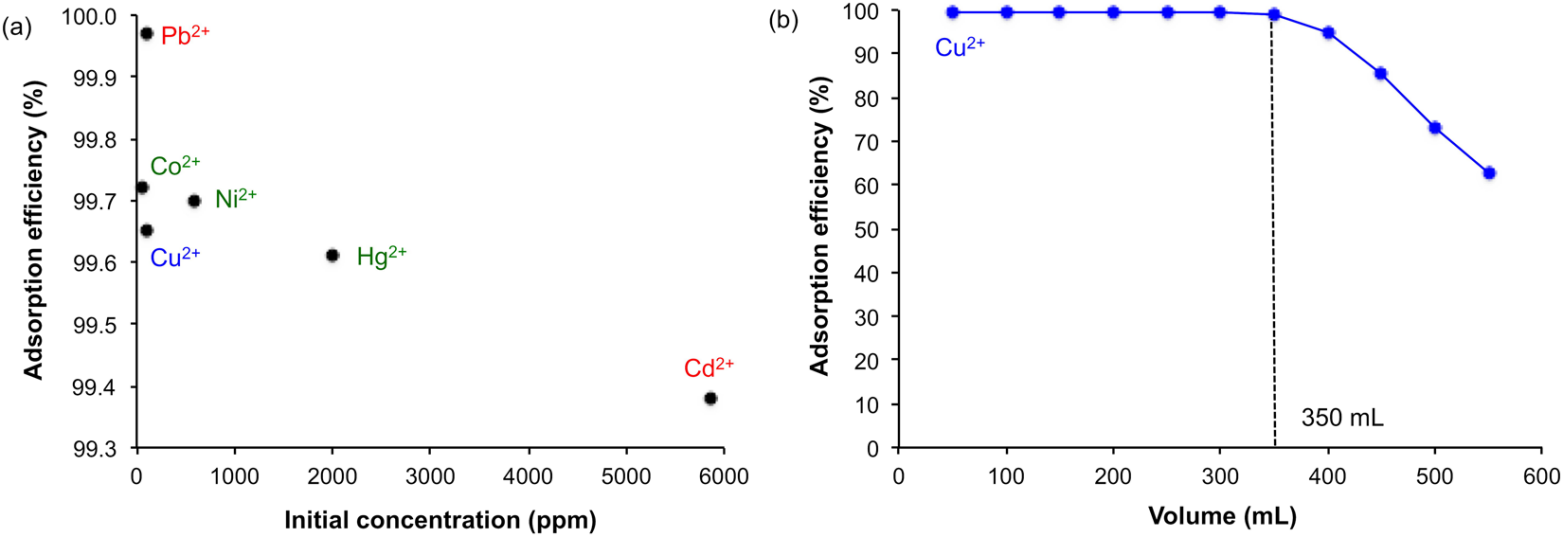
Uptake efficiency of the quartz wool supported-CNTs. Plots of (**a**) SENT adsorption efficiency of different metal ions as a function of initial concentration of 50 mL sample through 0.5 g SENT, and (**b**) change in adsorption efficiency as a function of solution volume for Cu^2+^ (100 ppm) showing the saturation point as defined by the volume above which the adsorption efficiency decreases.

While the adsorption efficiency is >99.4% for all metals, it appears that it is generally dependent on the initial concentration (C_i_), i.e. higher C_i_ results in lower sorption efficiency (Fig. 3a). However, it is worth noting that there is also an effect of the metal species, since Cu^2+^  < Co^2+^  < Pb^2+^ using similar C_i_. Also as seen from Fig. 3a there appears to be no dependence on the counter ion. To determine if the reason for the difference can be explained by the lower uptake being closer to the saturation, standard solutions were adsorbed through a known mass of SENT and aliquots measured (e.g. Fig. 3b) and the resulting saturation concentrations determined (Table 1). This shows that for each metal studied the adsorption efficiencies (Table 1) are far from saturation conditions, and any difference between metals is a function of the relative binding efficiency to the epoxide functionality.

**Figure 4:**
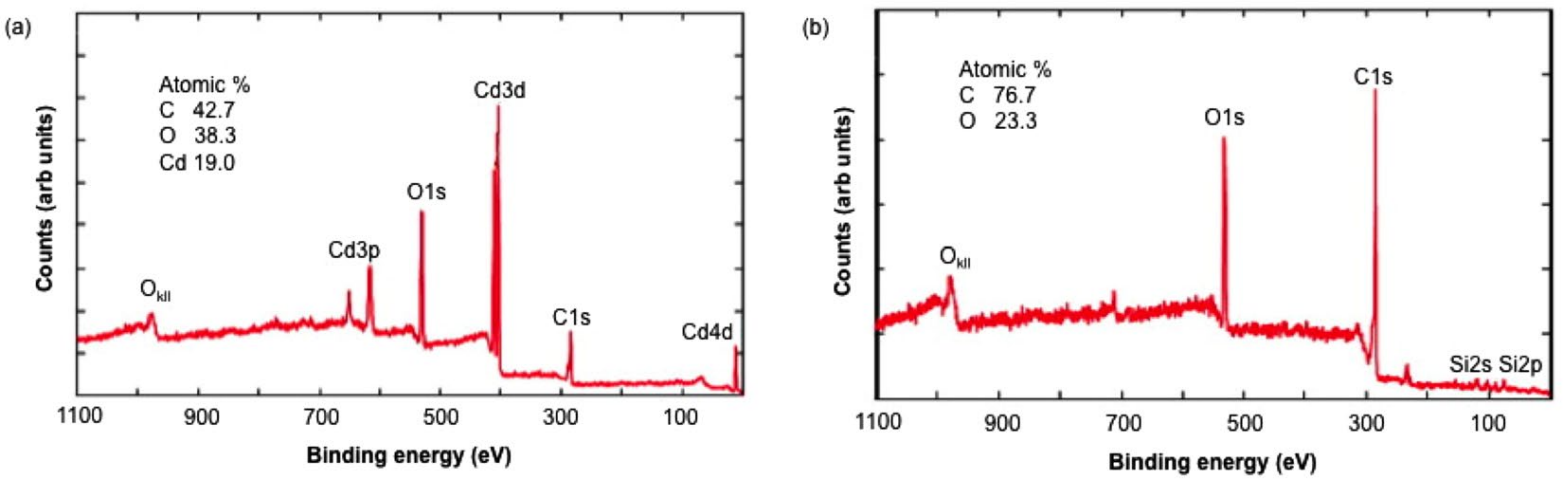
**X**-ray photoelectron spectra of the quartz wool supported-CNTs. XPS survey scans of (**a**) SENT after Cd^2+^ adsorption and (**b**) after washing with acetic acid:water (1:1) showing the removal of the Cd^2+^ and the underlying quartz (SiO_2_).

As noted in the introduction, it is desirable for an adsorbent to be able to be regenerated, not only to treat large volumes, but also provide a route to safely dispose or potentially recycle the heavy metals; many of which have commercial value. Firstly, washing the metal impregnated SENTs (M-SENTs) with DI water did not result in removal of any metal species (based upon XPS of the sample, and UV-visible spectroscopy of the washings); however, washing with a dilute solution (50%) of acetic acid showed removal of the metal. For example, Fig. 4 shows the XPS survey scans of Cd-SENTs before and after washing with aqueous acetic acid. Furthermore, a materials balance calculation of the atomic percentages of C and O before metal adsorption and after regeneration shows that there is a negligible loss of epoxide groups during the renewal process, ensuring recyclability. Metal adsorption after acetic acid treatment is not affected, i.e., the adsorption efficiency for a particular metal is retained.

## Discussion

It is worth comparing the maximum sorption capacities of SENT versus GO. Wang and co-workers have reported that GO can adsorb Cd (II) 106.3 mg/g^13^, in the present case we observe 581 mg/g, which compares with the far more complex cyanobacterium metallothionein decorated graphene (867 mg/g)^24^. We note that our results are dramatically different than those previously observed for nitric acid treated multi walled carbon nanotubes (MWCNTs), where only low-level adsorption of heavy metals (<5 mg/g) was observed^25^. In order to ascertain if the epoxide makes a significant difference, absorption of Cu(II) was measured using supported-CNTs after WAO treatment. Uptake was 1.6 mg/g consistent with the previous results on non-supported acid treated MWCNTs^25^. We propose that the lower levels of oxygen functionality observed with acid treatment^26^ versus epoxidation^15^ accounts for the exceptional improvement in metal uptake for the SENTs. Catalytic de-oxygenation of SENTs using ReMeO_3_/PPh_3_^14, 15^ indicates 48–63 CNT carbon atoms (C_CNT_) per oxygen depending on the sample. Unlike the equivalent measurement with epoxidized SWCNTs (C_CNT_:O = 5–9)^15^ the variation in the value in the present case is expected depending on the number of walls in the MWCNT in any given batch.

A key question is whether metal adsorption using SENT is a realistic approach to metal removal. As an extreme example, water wells in Koekemoerspruit (Africa) have been reported to contain 0.010 mg/L^26^, which far exceeds the WHO limit (0.003 mg/L)^27, 28^. It should be noted that even with these high levels an adsorption efficiency of > 70% would be acceptable. Given 1 g of SENT has the potential to adsorb 581 mg, that sample could treat 83,000 L of contaminated water down to WHO limits. Assuming 7.5 L per day per person as a minimum requirement, then 1 g SENT could treat sufficient water for 11,000 people.

Although acetic acid is a low cost bulk chemical, in every region of the planet the ability to produce alcohol is ubiquitous and hence by the use of natural acetic acid bacteria (AAB) its conversion to vinegar. Thus, the recycling of the filter is possible is possible even in the remotest locations. Time trials indicated that a scaled-up version of the filter (200x) could filter 5 L of water in 1 minute and be renewed in just 1.5 min; moreover, the 12-trial lead saturation analysis indicated that the filter could maintain a filtration capacity greater than 99.9% for up to 70 L/100 g of SENTs filter medium, before it needs to be renewed. Finally, in large-scale operation, the regeneration process can be used for reclamation of the metals, while in rural areas evaporation of the metal extract to a solid waste that can be combined by the community could be more safely disposed of. Finally, a preliminary cost analysis shows that the filter medium can be effectively and inexpensively implemented in a real world setting, with a cost under $0.25/g including materials and manufacturing costs. In contrast, GO is at present ca. $100/g, thus, SENTs offer a realistic alternative and the ready incorporation into traditional membrane systems offers the additional potential as an industrial process.

## Methods

### Materials

All materials were used as received unless otherwise noted. Acetic acid (≥99.7%), cadmium(II) acetate dihydrate (98%), 3-chloroperoxybenzoic acid (*m*-CPBA, 77% max), copper(II) sulphate pentahydrate (>98%), dichloromethane (≥99.5%), ferrocene (98%), lead(II) acetate trihydrate (>99%), mercury(II) chloride (99%), zinc(II) nitrate hexahydrate (98%) were purchased from Sigma-Aldrich, MeReO_3_ from Strem Chemical, toluene (99.98%) from OmniSolv EMD, HCl (1.0 N) from EMD Chemicals, cobalt(II) chloride hexahydrate (97%), nickel(II) chloride hexahydrate from Acros Organics, quartz wool (12 µm diameter), and Ar and H_2_(5%)/Ar high purity gases were purchased from Matheson TRIGAS. Triphenylphosphine (Aldrich) was recrystallized from EtOH.

### Synthesis of quartz supported epoxidized MWCNTs (SENTs)

A sample of quartz wool (3.45 g) was placed in the quartz reactor tube (34 mm ID), which was then inserted into a Nanotech Innovations SSP-354 tabletop horizontal tube reactor such that the quartz wool was positioned at the front of the growth zone to facilitate MWCNT growth using a modification of previously reported methods^16^. Ferrocene precursor solution (5 mL, 0.233 M in toluene) was drawn into a clean Hamilton syringe with a 0.26 mm diameter needle; then inserted into the reactor. The system was purged with 5% H_2_/95% Ar carrier flow gas at 1.5 L.min^−1^. The injection furnace was heated up to 225 °C, and the growth furnace heated up to 900 °C. Once heated, the precursor was injected at 3 mL/h. After the full volume of the precursor solution was injected, the system was cooled and the flow gas was stopped. The quartz-supported MWCNTs (ca. 6.95 g) were removed from the furnace and a small sample (100 mg) was placed in a ceramic combustion boat (7 mL) and positioned within a clean quartz tube in the Nanotech Innovations SSP-354 reactor. Air was flowed (145 mL/min) into the reactor via a Kontes bubbler filled with deionized water. The furnace was heated to 225 °C, and the quartz-supported MWCNTs were exposed to wet air at this temperature for 5 h^18^. After the furnace cooled down, the sample was removed and placed in a 250 mL round-bottom flask. To this was added HCl solution (100 mL, 1.0 N). The sample was sonicated for 15 min and then stirred for 5 h, followed by dilution with DI H_2_O (500 mL) and then filtered through a 0.45 μm polytetrafluorethylene (PTFE) membrane. The quartz-supported MWCNTs were washed with a large amount of DI water and dried in air for 4 h followed by 2 h at 200 °C. After cooling a sample of quartz-supported MWCNTs (40 mg) was added to a solution of *m*-CPBA (1.15 g) in CH_2_Cl_2_ (60 mL). The solution was stirred overnight and then filtered through a 0.45 μm PTFE membrane and washed with CH_2_Cl_2_ (2 × 30 mL)^15^. The quartz-supported epoxidized-MWCNTs (SENTs) were dried in vacuo at 80 °C for 3 h. The extent of epoxidation was quantified using the ReMeO_3_/PPh_3_ method previously reported^15^.

### Metal adsorption

In a typical protocol, a 100 mL burette cleaned consecutively using a KOH/isopropyl alcohol, tap water, and de-ionized water was, after drying, packed with SENTs (0.5 g). A solution (50 mL) of appropriate metal salt (Cd^2+^, 0.052 M; Cu^2+^, 0.052 M; Hg^2+^, 0.01 M; Ni^2+^ 0.01 M; Co^2+^, 0.001 M; Pb^2+^, 100 ppm) was then poured through the filter medium and aliquots collected at the bottom for UV-visible analysis. The procedure was conducted three times consecutively using the same filter medium with each metal. A time trial was conducted for each filtration trial to determine, on average, the speed of filtration in mL.min^−1^. XPS was performed directly after the metal filtration by selecting a small sample of the filter material to confirm metal uptake. A sample (ca. 5 mg) of the material immediately after the metal filtration was used for Raman spectral analysis (633 nm incident wavelength). In order to regenerate the filter and desorb the metal ion, the filter material was treated with 50 mL of 50:50 (by volume) de-ionized water/acetic acid, after which XPS analysis was performed. Time trials were conducted to determine how quickly the filter medium could be renewed. Subsequently, three more consecutive filtration trials of appropriate metal salt to confirm the renewal of the filter medium. For TGA analysis epoxidized MWCNTs (without the quartz substrate) were added to a standard solution of the appropriate metal salt. The solution was stirred overnight and then filtered through a 0.45 μm PTFE membrane. The CNTs were rinsed with a large amount of deionized water and dried in air for 4 h. followed by 2 h. in a at 200 °C.

### Renewal of SENTs

A sample of SENTs (20 mg) that had undergone the metal sorption process was added to the flask containing acetic acid in DI-H_2_O (150 mL, 50%). The reaction mixture was stirred overnight, and filtered through a 0.45 μm PTFE membrane. The acetic acid solution was retained and the SENT was washed with a large amount of DI-H_2_O before repeating the metal sorption process to compare the filtration capacity of the filter medium before and after the renewal.

### Characterization

Scanning electron microscopy (SEM) images were carried out with FEI Quanta 400 by placing samples on double-sided carbon tape that was fixed to aluminium SEM stubs. Images were acquired at a typical operating voltage of 20 kV, with a working distance of 10 mm, spot size 3 in Hi-VAC mode. Raman spectra were obtained using a Renishaw inVia Raman Microscope, at 633 nm, using a 50x LWD lens, data was acquired with 3 accumulations between 100 cm^−1^ and 3300 cm^−1^ with cosmic-ray background removal applied. The G’ peak was often the most prominent and so it was used to calibrate the laser spot to maximize signal intensity at the detector. X-ray photoelectron spectra (XPS) were acquired on a PHI 5700 ESCA system (Physical Electronics) at 15 kV, using an aluminium target and an 800 μm aperture. Samples were pressed into indium metal. UV-visible spectra were collected on a Cary 100 spectrophotometer, scanning between 550 and 900 nm with a step size of 0.5 nm.
